# Correction: MicroRNA-613 inhibits cell growth, migration and invasion of papillary thyroid carcinoma by regulating SphK2

**DOI:** 10.18632/oncotarget.28155

**Published:** 2022-04-11

**Authors:** Wangwang Qiu, Zhili Yang, Youben Fan, Qi Zheng

**Affiliations:** ^1^Department of General Surgery, Shanghai Jiao Tong University Affiliated Sixth People’s Hospital, Shanghai 200233, P.R. China; ^*^These authors contributed equally to this work

**This article has been corrected:** In Figure 5D, the miR-613+pcDNA3.1/SphK2 image in the ‘migration’ row contains an accidental overlap of the miR-613 image in the ‘invasion’ row. The corrected Figure 5, produced using the original data, is shown below. The authors declare that these corrections do not change the results or conclusions of this paper.

Original article: Oncotarget. 2016; 7:39907–39915. 39907-39915. https://doi.org/10.18632/oncotarget.9530

**Figure 5:**
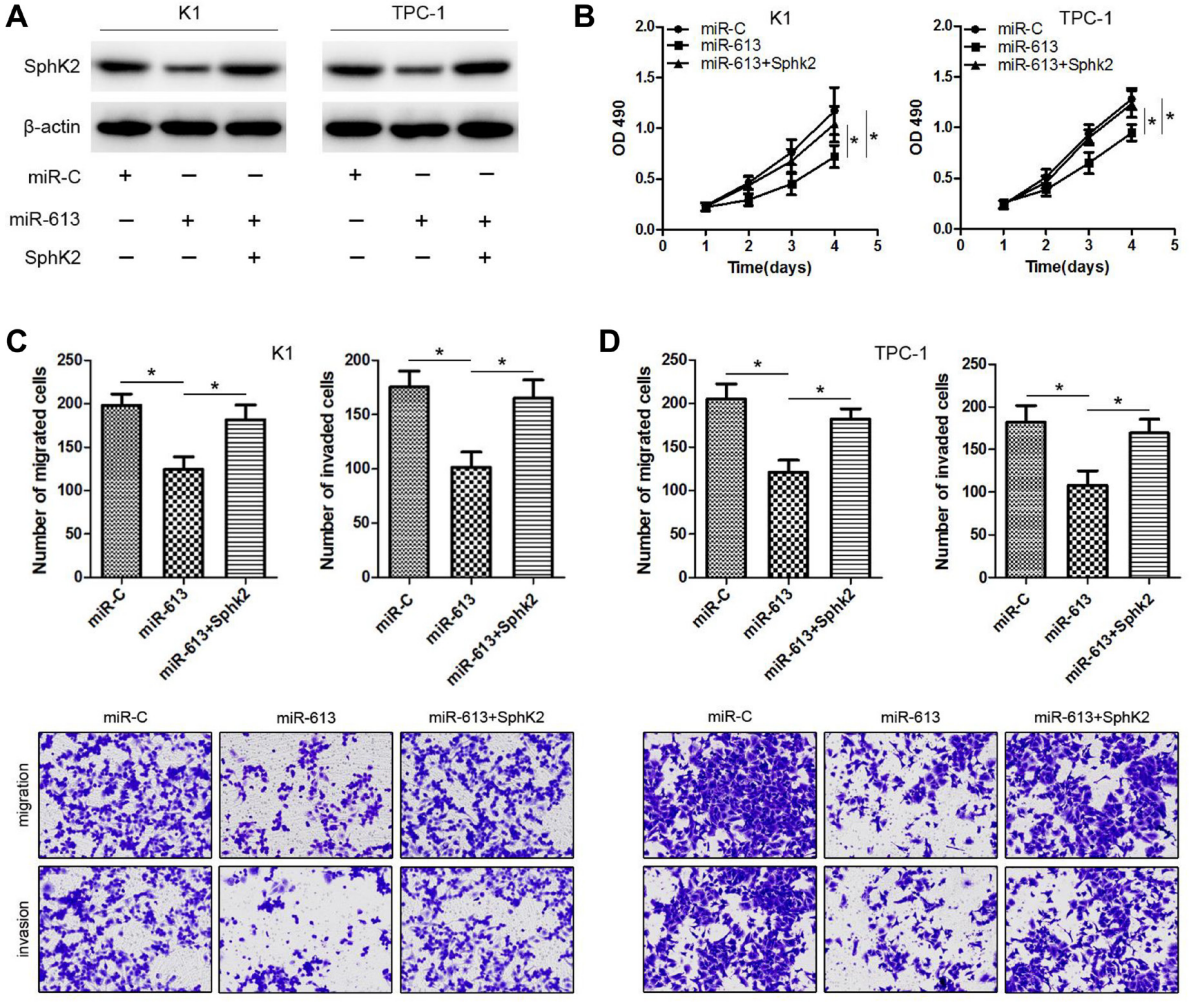
Ectopic expression of *SphK2* restores the effects of miR-613 on cell proliferation, migration and invasion in PTC cells. K1 and TPC-1 cells were respectively co-transfected with miR-613 and *SphK2* ORF without the 3′-UTR. (**A**) *SphK2* expression was measured using western blots for each group of transfected K1 and TPC-1 cells. (**B**–**D**) Cell proliferation by MTT assays, migration capacity by colony formation assays, and invasion capacity by transwell assays. ^*^ *P* < 0.05.

